# Effects of Polyvinyl Chloride Microplastics on the Reproductive System, Intestinal Structure, and Microflora in Male and Female Mice

**DOI:** 10.3390/vetsci11100488

**Published:** 2024-10-09

**Authors:** Yang-Kai-Xin Yang, Shu-Jun Ge, Qi-Ling Su, Jin-Jun Chen, Jiang Wu, Kai Kang

**Affiliations:** 1 Department of Veterinary Medicine, College of Coastal Agricultural Sciences, Guangdong Ocean University, Zhanjiang 524088, China; yangkaixinyang37@126.com (Y.-K.-X.Y.); geshujun2024@126.com (S.-J.G.);; 2 Department of Animal Science, College of Coastal Agricultural Sciences, Guangdong Ocean University, Zhanjiang 524088, China

**Keywords:** polyvinyl chloride, microplastics, male and female mice, intestine, reproductive system

## Abstract

**Simple Summary:**

The aim of this experiment was to investigate the distinct effects of microplastic-polyvinyl chloride (PVC) on the reproductive system, intestinal tissue structure, and microbial flora in male and female mice. The impact of PVC microplastics on intestinal microflora differed between males and females, showing contrasting trends. Both male and female mice exhibited impaired structural integrity of the intestine and reproductive systems due to polyvinyl chloride microplastics. Additionally, there was a significant correlation between disturbances in intestinal microflora caused by PVC microplastics and reproductive system function.

**Abstract:**

The pervasive use of plastics in numerous industrial sectors has resulted in the circulation of microplastics across diverse ecosystems and food chains, giving rise to mounting concerns regarding their potential adverse impacts on biological systems and the environment. The objective of this experiment was to investigate the distinct effects of microplastic-polyvinyl chloride (PVC) exposure on the reproductive system, intestinal tissue structure, and intestinal microbial flora of both male and female mice. A total of 24 4-week-old Kunming mice were randomly assigned to one of four groups: male control group (CM), female control group (CF), male PVC test group (PVCM), and female PVC test group (PVCF) (n = 6). The findings revealed that in terms of the reproductive system, the PVCM group exhibited an impaired testicular structure with an irregular arrangement and a significant reduction in spermatogonia, spermatocytes, and spermatozoa within the seminiferous tubules (*p* < 0.01). The PVCF group exhibited a notable decrease in ovarian follicles (*p* < 0.01), accompanied by a reduction in uterus volume, fallopian tube volume, and muscle layer thickness, all of which also decreased significantly (*p* < 0.01). In comparison to the control groups, exposure to PVC resulted in a reduction in the width and height of the intestinal villi, accompanied by an increase in crypt depth. This led to a significant alteration in the ratio of villus height to crypt depth (V/C) (*p* < 0.01). Moreover, a reduction in microbial species diversity was observed within both the PVCM and PVCF groups; additionally, it was accompanied by contrasting changes in relative abundance and functional gene profiles among the major intestinal flora constituents. In summary, the findings indicate that PVC induces damage to both male and female mice reproductive and digestive systems, further exhibiting notable sex-dependent effects on mouse intestinal microflora composition, which correlates significantly with its impact on reproductive organs.

## 1. Introduction

In 2004, Thompson et al. identified the presence of plastic particles measuring less than 5 mm in diameter in marine waters and sediments. Microplastics are persistent organic pollutants that are widely distributed in nature and exhibit greater resistance to degradation compared to general plastics [1]. Polyvinyl chloride (PVC) is a polymer material obtained through the free radical polymerization of vinyl chloride monomer, with polyvinyl chloride representing its primary component. The accumulation of PVC within organisms can occur through food consumption, with the substance becoming enriched across various organisms within the food chain and participating in ecological cycles [2]. Due to their small size, microplastic particles have been found to be present in the digestive system, respiratory system, reproductive system, and circulatory system of organisms; this presents a significant potential risk for environmental harm [3].

Experimental evidence has demonstrated that microplastics can accumulate in the testes and ovaries, resulting in the development of inflammation and oxidative damage to these glands and germ cells [4]. The accumulation of microplastics in the testes of mice has been demonstrated to results in reproductive toxicity, with a significant decrease in sperm count and motility, as well as a notable increase in the rate of sperm deformity. Furthermore, microplastics have been observed to affect the interstitial looseness of the offspring mice’s testes, the exfoliation of the spermatogenic epithelium, and the reduction of sperm number within the lumen. These alterations in the testicular histology of the offspring mice result in disorders of spermatogenesis. With regard to the ovaries, exposure to microplastics has been observed to increase the rate of atresia in follicles, impede follicular maturation, and induce reproductive toxicity [5]. Liu et al. observed the accumulation of microplastics in the intestines, ovaries, and uterus of exposed mice [6]. Moreover, a study on BALB/C female mice demonstrated that microplastics not only impair the uterus, but also affect immune function within the reproductive system. This presents a challenge for animal breeding [7].

The primary exposure pathways of microplastics to organisms primarily encompass ingestion, inhalation, and dermal contact [8]. In the event that microplastics enter the body through food consumption, the intestinal barrier plays a pivotal role in impeding their migration into other tissues. The integrity of this barrier is essential for optimal nutrient absorption, which is vital for animal growth and reproduction. Alterations in the structure and microbiota of the intestines induced by microplastics can disrupt normal digestive system function, leading to systemic diffusion and transport of microplastics and the potential development of disease [9]. At present, there is growing interest in the study of the gastrointestinal tract and its associated microbiota. However, research on the biological toxicity of microplastics has predominantly focused on aquatic animals and soil organisms, with only a limited number of studies conducted on mammals. For example, after 30 days of exposure to polyvinyl chloride particles (PVC), approximately 67% of healthy adult fish from Norway exhibited moderate damage to their intestinal tissue compared to the control groups. Following a 90-day exposure period, approximately 50% of the subjects exhibited severe damage characterized by an edematous serosa layer and mucosal muscle layer, along with evident vasodilation and leukocyte infiltration. This indicates that chronic exposure to microplastics can induce lesions in the intestinal tissue, accompanied by inflammatory responses [10]. SARKER et al. demonstrated that exposure to microplastics results in intestinal damage and metabolic abnormalities in terrestrial organisms, thereby influencing the mutual relationship between intestinal flora and disease. Intestinal damage results in alterations in the composition of the intestinal flora, and the presence of certain intestinal bacteria can also cause damage to the intestine. It is, therefore, evident that changes in the intestinal microflora caused by microplastics have a deleterious effect on animal growth [11,12,13]. Furthermore, Botterell et al. have corroborated the negative impact of microplastics on animal growth and reproduction [14]. With the advent of advanced techniques in intestinal genomics, metabolomics, and proteomics, research has uncovered a spectrum of dysbiosis and shifts in microbial composition within the human gut microbiome, which is linked to a range of diseases, including inflammatory bowel disease, type 1 diabetes, and rheumatoid arthritis [15]. Animal intervention studies have also indicated that particular gut bacteria may facilitate disease progression via their metabolites.

The reason why microplastics cause animal growth and reproduction obstacles is closely related to their impact on the reproductive system and the intestine. In this experiment, we observed the effects of commonly used PVC microplastics on the testis, ovary, uterus, fallopian tube, small intestine, and intestinal microflora of male and female mice by were observed incorporating them into their feed. The objective of this study was to examine potential sex-based differences in the impact of PVC microplastics on mice, and to gather data on the mechanisms underlying the effects of foodborne microplastics on reproductive function and intestinal health in mammals of different sexes. This will facilitate a more comprehensive assessment of the harmful effects of microplastics on mammals.

## 2. Materials and Methods

### 2.1. Experimental Animals and Grouping Treatment

A total of 24 30-day-old Kunming mice (12 males and 12 females) were used. The mice were randomly divided into 4 groups (n = 6), which were marked as control group male mice (CM group), control group female mice (CF group), PVC group male mice (PVCM group), and PVC group female mice (PVCF group). The control group was fed normal chow, and the PVC group was fed chow containing 30 g·kg^−1^ microplastics PVC for 28 days. In the experiment, mice were fed and managed by each group, and each mouse independently ate about 7 g of self-made mouse food per day, which indicated that mice in the PVC group consumed 0.21 g of microplastics per day. All mice were kept in an environmentally controlled room; the temperature was maintained at (25 ± 1) °C, and the relative humidity was (65–85)%.

All experimental protocols were approved by the Animal Ethics Committee of Guangdong Ocean University (IACUC No. GDOU-LAE-2020-007), and the experiment was performed according to the ethical guidelines of the European Community guidelines.

### 2.2. Production of Mouse Cake

The raw materials required for the production of mouse food are fully mixed, and PVC microplastics are added (The specific formulation of the mice diet can be found in the article published by Su et al., from our research group [16]). In the PVC group, PVC microplastics were added to mouse food (purchased from Mason Biotechnology Co., Ltd., Dublin, Ireland, and the microplastics were screened using 600 mesh sieves before use), and the control group did not contain microplastics.

Plastic PVC powder was purchased from Meisen Biological Company (Xi’an, China). The microplastics were obtained using a 600-mesh screen (the diameter of PVC < 25 μm).

### 2.3. Paraffin Section and HE Staining of Mouse Reproductive System

After a feeding period of 28 days, the mice were euthanized by cervical dislocation; ovary, uterus, and fallopian tube tissues of female mice and testicular tissues of male mice were extracted. After washing with normal saline, the collected reproductive system tissues of the mice were immediately fixed in formalin for 12 h and then replaced with fresh formalin for 12 h. The samples were dehydrated with gradient concentration of alcohol, embedded in wax, sliced (thickness of about 4 μm), routinely stained with hematoxylin and eosin, microscopically examined, and image acquisition and analysis.

### 2.4. HE Section Analysis of Reproductive System in Male and Female Mice

Image J software 1.53e was used to repeatedly measure and calculate the slice image data of each reproductive system of male and female mice. The thicknesses of the epithelium and myometrium of the uterus and fallopian tube sections in the CF group and PVCF group were measured in female mice, and the follicles at all levels in the ovary were counted and measured. The inner diameter of each seminiferous tubule and the thickness of the boundary membrane between them were measured in the testicular sections of the CM group, and the PVCF group, and the spermatogonia, spermatocytes, and spermatozoa were counted.

### 2.5. Collection of Intestinal Microbial Samples in Mice and Paraffin Sections and HE Staining of Intestinal Tissues

After 28 days of feeding, mice were euthanized by cervical dislocation. The jejunum, cecum, and colon tissues of the mice were selected to collect intestinal contents, which were transferred to a sterile EP tube (1.0 ± 0.5 g/tube) and then placed on ice, frozen in liquid nitrogen and stored at −80 °C for intestinal microbiome analysis.

The duodenum, jejunum, and ileum tissues of male and female mice were collected at 5 mm each. After washing with normal saline, they were fixed in an EP tube containing 4% formaldehyde fixative, frozen in liquid nitrogen, and stored at −80 °C for subsequent intestinal sectioning and histological structure observation. After washing with normal saline, the collected reproductive system tissues of the mice were immediately fixed in formalin for 12 h and then replaced with fresh formalin for 12 h. The samples were dehydrated with a gradient concentration of alcohol, embedded in wax, sectioned with a low-temperature thermostat (thickness of about 4 μm), routinely stained with hematoxylin-eosin, microscopically examined, and image acquisition and analysis.

### 2.6. HE Section Analysis of Small Intestine in Male and Female Mice

Image J software 1.53e was used to measure and calculate the four data points for each segment of the small intestine slice image of male and female mice, and the local damage to the small intestine and the change in villus width were marked in the image. The control group and the PVC experimental group were compared, and the villus width, villus height, crypt depth, and other data of the small intestine of each group of male and female mice were measured, and the ratio of villus height to crypt depth (V/C) was obtained. The mean and variance of each group of data were calculated using Microsoft Excel 365 software, and the two decimal points were retained and expressed as the mean ± error. ▲: Indicates the position of intestinal villi in mice in the normal group and the PVC group. 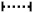: Indicates the width of intestinal villi measured in male and female mice (the same picture identification can be found in the article by our research group by Su et al. [16]).

### 2.7. Intestine Microbiome Analysis

Total DNA was extracted from intestinal content samples using the DNeasy PowerSoil Kit (Code No. 47014, QIAGEN, CA, Hamburg, Germany). Quantification was performed using a NanoDrop One spectrophotometer (NanoDrop Technologies, Wilmington, DE, USA) and a Qubit 3.0 Fluorometer (Life Technologies, Carlsbad, CA, USA). Specific primers with barcodes were used to amplify the V3-V4 region of the bacterial 16S rRNA gene by PCR. The primer pairs used were 341F (5′-CCTACGGGNGGCWGC) and 805R (5′-GACTACHVGGGTWTCTAATCC). Library preparation and sequencing were carried out using the VAHTS@ Universal DNA Library Prep Kit for Illumina V3 with VAHTS DNA Adapters set3-set6 for Illumina (Code No. ND607 and N806, NVIDIA, Santa Clara, CA, USA) provided by the Benagen Corporation (Wuhan, China), following official tutorials. PCR products underwent purification using magnetic beads, and size detection of the amplified products was conducted via Qubit analysis and agarose gel electrophoresis. After pooling based on read numbers and repairing pooled product ends accordingly, sequencing splice connections took place, followed by magnetic beads and quantification through Qubit analysis. Once quality control checks were passed at the library level, the completion stage was reached before high-throughput sequencing was executed using an Illumina NovaSeq 6000 sequencer (Illumina, San Diego, CA, USA).

### 2.8. Analysis of Small Intestinal Microbial Sequencing Data in Male and Female Mice

After barcode recognition and resolution of the samples, the abundance of amplicon sequence variants (ASVs) in the sample was analyzed using the DADA2 method, and QIIME2 (version 2022.3) software was used to filter low-abundance ASVs. Using the Silva database and the UNITE database as a reference, the Naive Bayes species classifier was constructed using the classify-sklearn algorithm of QIIME2, the species abundance at each level was analyzed, and a histogram was constructed. ACE, Shannon, Chao1, and other indices were used to evaluate the alpha diversity of the community. Beta diversity analysis was based on the OTU table for principal co-ordinates analysis (PCoA), and the Bray−Curtis algorithm was used to calculate the R^2^ value and *p* value to test the differences in species diversity among samples. Then, PLS-DA analysis was used to determine the degree of difference and similarity between groups and samples. The species composition of each group was analyzed based on the classification level. PICRUSt2 was used to analyze gene function in the samples. The data on intestinal microbiome were analyzed using Microsoft Excel 365 software. The data for each group are expressed as the mean ± error, and the TTEST function was used for multiple comparison tests.

Additionally, PICRUSt2 from Langille Lab was used to analyze gene function in KEGG (Kyoto Encyclopedia of Genes and Genomes) and COG (Clusters of Orthologous Groups of Proteins) databases. *t*-tests were employed to identify significant differences between groups with a significance level set at *p* < 0.05.

### 2.9. Statistical Analysis

Microsoft Excel 365 software was used to conduct preliminary statistics on the slice image data of each reproductive system of male and female mice. The data of each group are expressed as mean ± error, and the *t*-test was used to analyze the difference significance, and the test level was *p* < 0.05. In the table, different letters between the numbers in the same column or in the same line indicate a significant difference (*p* < 0.05), and uppercase letters indicate significance level A = 0.05; lowercase letters indicate significance level a = 0.01, and the same letter or no letter indicates no significant difference (*p* > 0.05).

The Bonferroni, multiple comparison test of GraphPad Prism 9 software, was used to perform two-way analysis of variance on the small intestinal slice data of male and female mice. A *t*-test was used to determine significant differences between the HE sections of the reproductive system of male and female mice. After the results of the statistical analysis were obtained, a histogram was constructed using GraphPad Prism 9 to show the differences in the data of each group. The significance level was set at *p* < 0.05.

Spearman correlation analysis was used to analyze the relationship between the level of intestinal microflora and function and the measurement data of the reproductive system by using the OmicStudio tool provided by https://www.omicstudio.cn/tool (accessed on 7 June 2024). A significance level of *p* < 0.05 was considered statistically significant, and the trend with a significant trend was defined as 0.05 ≤ *p* < 0.10.

## 3. Results

### 3.1. The Effect of Plastic PVC on the Structure of Reproductive System in Male and Female Mice

#### 3.1.1. Effects of Microplastic PVC on the Morphology of Gonadal Organs in Male Mice

**Figure 1:**
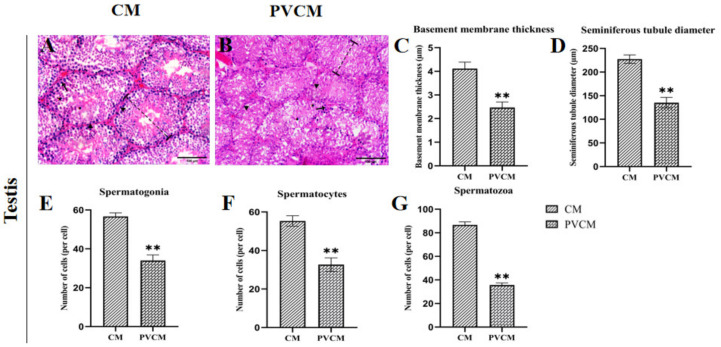
Histological sections and microstructural analysis of mouse testes. (**A**) Testicular group of CM, (**B**) testicular group of PVCM, (**C**) thickness of the boundary film, (**D**) inner diameter of convoluted tubules, (**E**) spermatogonia count, (**F**) spermatocyte count, and (**G**) spermatozoa count. CM, Control group of males; PVCM, PVC test group of males. ▲: Indicates where the boundary film is located. 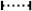: Indicates the diameter of the tubule of the testes measured in the CM and PVCM groups. ↑: Indicates the location of spermatogonia. ★: Indicates the position of the spermatocytes. ●: Indicates the location of spermatozoa. Data are presented as mean ± SD. **, *p* < 0.01. Scale bar = 100 μm.

The morphology of the seminiferous tubules in the testes of male mice in the control group was mostly regular, oval, or round. The seminiferous tubules were closely connected, and spermatogonia were attached to the basement membrane. It can be seen that the cells at all levels were arranged from the periphery to the lumen, and the sperm cells were dense. There was a large number of shaped sperm in the lumen, and the testicular stroma was clear (Figure 1A). Compared to the control group, the intercellular connections in some seminiferous tubules of the PVC group were loose and disorderly arranged (Figure 1B). The boundary membrane between the seminiferous tubules of each koji group was significantly reduced (*p* < 0.01) (Figure 1C). The inner diameter of a single seminiferous tubule was significantly reduced (*p* < 0.01) (Figure 1D) after PVC exposure. The development and growth of spermatogonia, spermatocytes, and spermatozoa were inhibited and this number was significantly reduced (Figure 1E–G).

#### 3.1.2. Effect of Microplastic PVC on the Morphology of Reproductive Gland Organs in Female Mice

**Figure 2:**
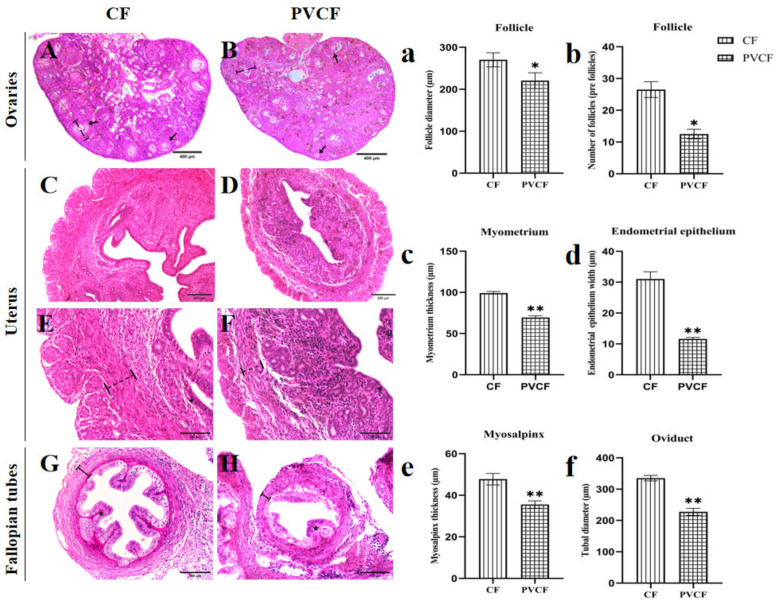
Histological sections and microstructural analysis of the mouse ovary, uterus, and fallopian tubes. (**A**) Ovarian group of CF; (**B**) ovarian group of PVCF; ●: Indicates the location of the follicle. 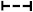: Indicates the follicle diameter of the ovary measured of CF and PVCF. ↑: Indicates the follicle. (**C**) Uterus group of CF; (**D**) uterine group of PVCF; (The same magnification figure (**B**–**D**) shows that the uterus is significantly reduced under PVC exposure) (**E**) uterine group of CF; (**F**) uterine group of PVCF; ▲: Indicates the position of the uterine epithelium. 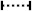: Indicates the thickness of the muscle layer of the uterus measured. (**G**) Fallopian tube group of CF; (**H**) Fallopian tube group of PVCF; ★: Indicates the position of fallopian tube epithelium. 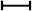: Indicates the muscular thickness of the fallopian tube measured. (**a**) Follicle diameter of CF and PVCF; (**b**) number of follicles of CF and PVCF; (**c**) myometrium thickness of CF and PVCF; (**d**) thickness of uterine epithelial cells of CF and PVCF; (**e**) Thickness of musculature of tubal tube; (**f**) diameter of tubal lumen; Data are presented as mean ± SD. *, *p* < 0.05; **, *p* < 0.01. Scale bar = 400 μm in (**A**,**B**); 200 μm in (**C**,**D**); 100 μm in (**E**–**H**).

Ovarian tissues were analyzed by HE staining. For the measurement of diameter, mature follicles were mainly measured, and only the total number was considered. The uterus and ovaries belong to the same cycle, but all mice were sampled at 58 days of age. Mice in the normal control group and the PVC group were located in the same estrus cycle, and they could be compared. The ovaries of the CF group showed normal development of follicles and corpus luteum at different levels, and the structure between different follicles showed clear and normal development (Figure 2A). In the PVCF group, the number of corpora lutea decreased, the number of atretic follicles increased, the diameter of follicles decreased significantly (*p* < 0.05) (Figure 2a), and the number of follicles at all levels decreased significantly (*p* < 0.05) (Figure 2b).

Compared with the PVCF group, the endometrium of the CF group was complete, with a clear tissue structure, abundant blood vessels and glands in the endometrium, and the morphology of glands was normal. The columnar epithelial cells of the mucous membrane are normal, single-layer columnar, arranged neatly and closely. In the PVCF group, the uterus was significantly smaller, the endometrial tissue was densified, the nucleus per unit area increased, and endometrial folding was significantly reduced (Figure 2D). The myometrium was significantly thinner (*p* < 0.01) (Figure 2c), the longitudinal muscle tissue of the uterus was loose, the columnar epithelial cells of the mucosa were significantly reduced (*p* < 0.01) (Figure 2d), and the structure was not clear.

Compared with the CF group, the number of folds in the fallopian tube sections of the PVCF group was reduced, the normal structure of the fallopian tube serosa and mucosa was destroyed, the single-layer columnar cells were arranged in disorder, and the morphological changes were irregular (Figure 2H). The connection between the mucosal layer and the muscular layer was not close; the muscular layer was significantly thinner (*p* < 0.01) (Figure 2e), and the lumen diameter was significantly reduced (*p* < 0.01) (Figure 2f).

#### 3.1.3. Association Analysis of Intestinal Microflora and Reproductive System Tissue and Organ Indexes in Male and Female Mice

**Figure 3:**
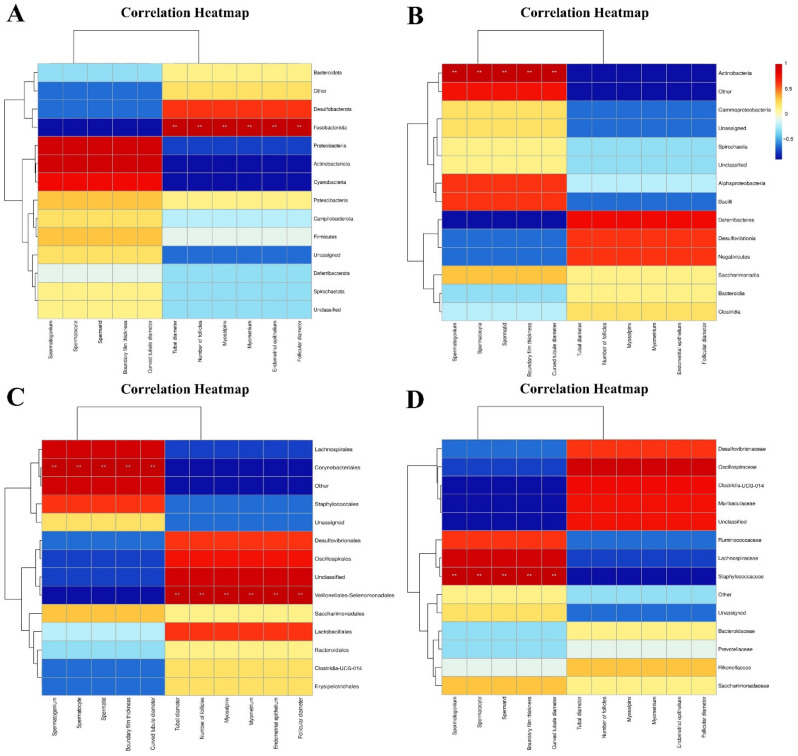
Spearman correlation analysis of intestinal microflora and reproductive system of male and female mice in the PVC group at the phylum, class, order, and family levels. (**A**) Phylum horizontal correlation analysis of females and males; (**B**) class horizontal correlation analysis of females and males; (**C**) order horizontal correlation analysis of females and males; and (**D**) family horizontal correlation analysis of females and males. **, *p* < 0.01.

After Spearman correlation analysis, the results showed that at the phylum level, *Fusobacteriota* was positively correlated with the measurement data of fallopian tube diameter and muscle layer thickness, uterine muscle layer and epithelial thickness, ovarian follicle number and diameter in the reproductive system of female mice (*p* < 0.01) (Figure 3A). At the class level, *Actinobacteria* was positively correlated with the thickness of the boundary membrane of the testis, diameter of the seminiferous tubules, and number of spermatogonia, spermatocytes, and sperm (*p* < 0.01) (Figure 3B). At the order level, *Corynebacteriales* was positively correlated with the above measurement data of male testes (*p* < 0.01), while *Veillonellales-Selenomonadales* was positively correlated with the above measurement data of the female reproductive system (*p* < 0.01) (Figure 3C). At the family level, *Staphylococcaceae* was positively correlated with the above measurement data of male mice testes (*p* < 0.01) (Figure 3D).

### 3.2. Effects of Microplastic PVC on the Structure of Small Intestine in Male and Female Mice

**Figure 4:**
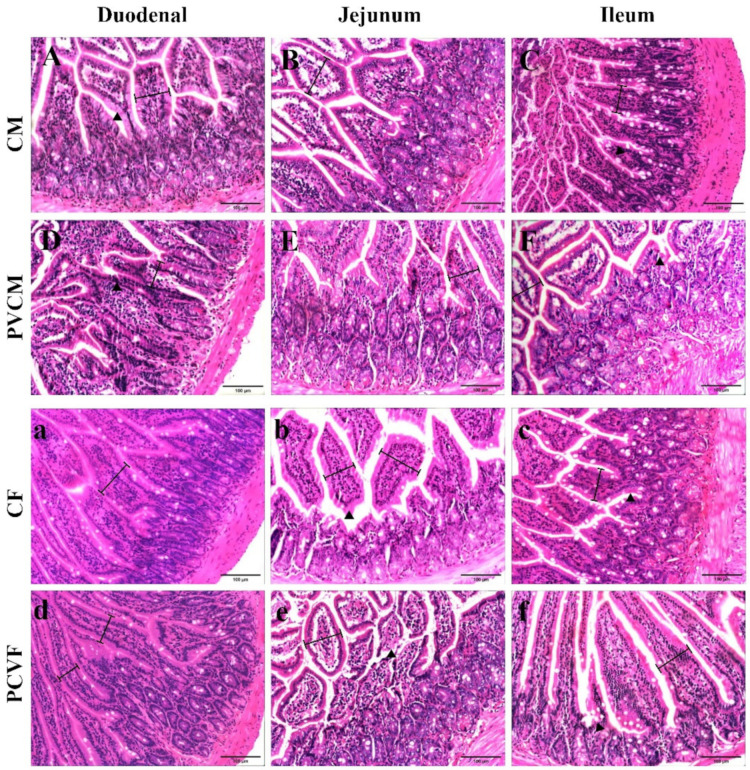
Intestine section of mice exposed to microplastics. (**A**) Duodenal group of CM; (**B**) jejunum group of CM; (**C**) ileum group of CM; (**D**) duodenum group of PVCM; (**E**) jejunum group of PVCM; (**F**) ileum group of PVCM; (**a**) duodenal group of CF; (**b**) jejunum group of CF; (**c**) ileum group of CF; (**d**) duodenum group of PVCF; (**e**) jejunum group of PVCF; (**f**) ileum group of PVCF; ▲: Indicates the position of intestinal villi of mice in the normal group and the PVC group. 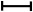: Indicates the width of intestinal villi measured in male and female mice. Scale bar = 100 μm.

In the PVCM group, compared with the CM group, the small intestine structure was changed, local villi were destroyed, villus structure was changed (Figure 4D,F), and villus epithelium became thinner. The intestinal villus width of the jejunum was significantly decreased (*p* < 0.05). The villus height and crypt depth of the duodenum, jejunum, and ileum in the PVCM group were significantly lower than those in the control group (*p* < 0.05). The ratio of villus height to crypt depth (V/C) in the ileum was significantly reduced (*p* < 0.05) (Table 1).

In the PVCF group, the local villus structure was destroyed (Figure 4e,f), and the villus epithelial cells atrophied and became smaller in sections of the small intestine. The width of the intestinal villi in the jejunum and ileum significantly decreased (*p* < 0.05). Compared with the control group, the villus heights of the duodenum, jejunum, and ileum were significantly decreased (*p* < 0.05). Compared with the control group, the V/C values of ileum were significantly decreased (*p* < 0.05), and jejunum was significantly decreased (*p* < 0.05). Compared to the duodenum and jejunum, the microstructure of the mouse ileum was most affected by microplastics (Table 1).

### 3.3. Taxonomic Analysis of Intestinal Microflora Characteristics of Male and Female Mice

**Figure 5:**
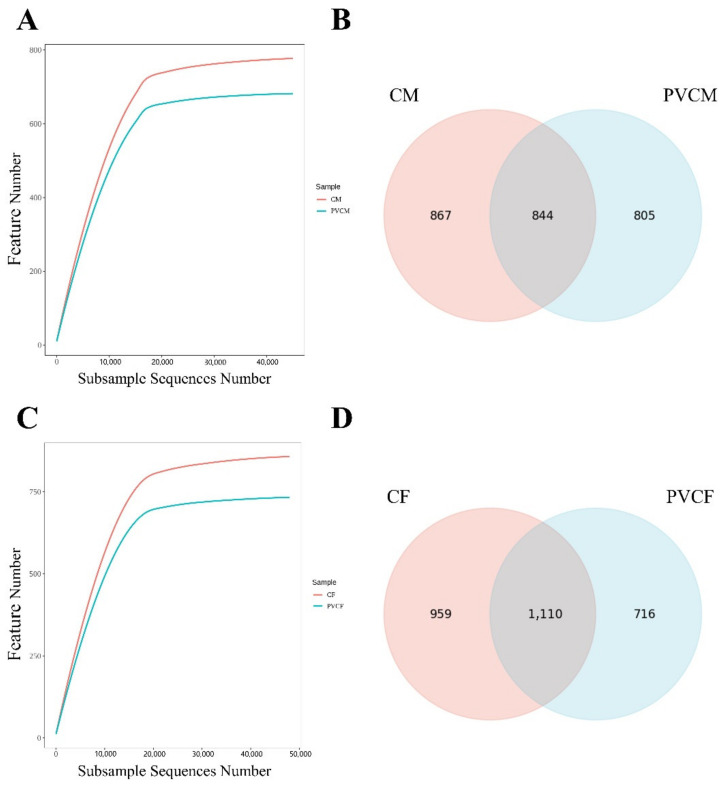
Alpha diversity analysis and common feature analyses of the gut microbial community of mice exposed to microplastics. (**A**) Intergroup dilution curve of CM and PVCM; (**B**) common characteristic Venn diagram of CM and PVCM; (**C**) intergroup dilution curve of CF and PVCF; and (**D**) common characteristic Venn diagram of CF and PVCF.

By 16 S rRNA sequencing, an average of 114,531 valid sequences were generated for each sample in the PVCF group and an average of 116,383 valid sequences were generated for each sample in the PVCM group. The curve tends to be flat in the dilution curve, indicating that the sample sequence is sufficient and data analysis can be performed (Figure 5A,C). Comparing the CF group with the PVCF group, the number of unique features in the PVCF group was 716, the number of unique features in the CF group was 959, and the number of common features between the groups was 1110 (Figure 5D). Comparing the CM group with the PVCM group, the number of unique features in the PVCM group was 805, the number of unique features in the CM group was 867, and the number of common features between the groups was 844 (Figure 5B). The change in RNA levels in female mice was lower than that in male mice.

Female and male mice were used as research objects to observe the effects of PVC on the number of intestinal microbial species in male and female mice at the level of phylum, class, order, family, genus, and species under microplastic exposure. Compared with the control group, except for the family and genus levels, the microbial species in the PVCF group increased at all levels, but the differences were not significant. Compared with the control group, except for the order and family levels of PVCM, the microbial species at the phylum, class, genus, and species levels decreased, but they were not significant (Table 2).

### 3.4. Analysis of Species Composition of Intestinal Microflora in Male and Female Mice at Different Levels

In order to explore the effect of PVC microplastics on the species composition of intestinal microflora in mice of different classification groups, we analyzed the species composition of intestinal microflora in mice at all levels. PVC microplastic treatment significantly affected the abundance of gut microbiota at different levels. The effect of PVC microplastics on intestinal microflora in mice was discussed, and sex was used as the classification standard.

**Figure 6:**
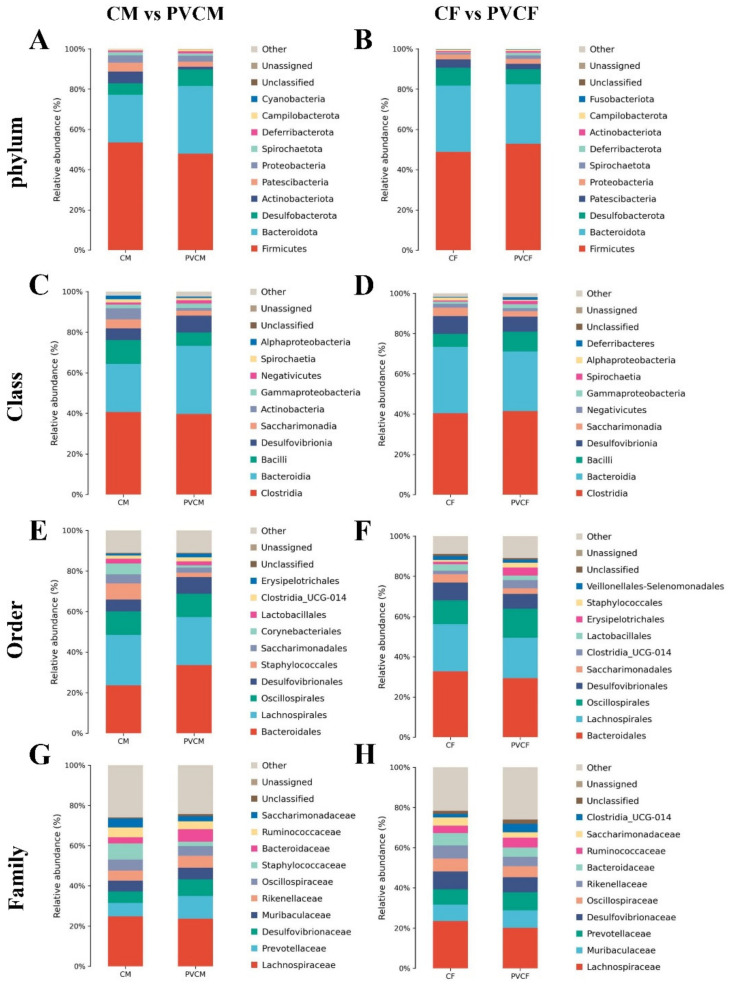
Horizontal species composition bar charts of the species of gut microbiota in mice exposed to microplastics. (**A**) Phylum horizontal of CM and PVCM; (**B**) Phylum horizontal of CF and PVCF; (**C**) Class horizontal of CM and PVCM; (**D**) Class horizontal of CF and PVCF; (**E**) Order horizontal of CM and PVCM; (**F**) Order horizontal of CF and PVCF; (**G**) Family horizontal of CM and PVCM; (**H**) Family horizontal of CF and PVCF.

In male mice, the four most important microbial phyla in the intestine are *Firmicutes*, *Bacteroidetes*, *Desulfobacteria*, and *Proteobacteria*. At the phylum level, the relative abundance of *Bacteroidetes* and *Desulfobacteria* increased. The relative abundances of *Actinobacteria*, *Firmicutes*, and *Proteobacteria* decreased significantly (Figure 6A). At the class level, among the four most important microbial classes in the intestinal tract of PVCM, the relative abundance of *Desulfobacteria* and *Bacteroidetes* increased, and the relative abundance of *Bacilli* and *Clostridium* decreased (Figure 6C). At the order level, the relative abundances of *Desulphurales* and *Bacteroideales* increased, while the relative abundances of Trichospirillales and Tremillales decreased (Figure 6E). At the family level, the relative abundance of *Bacteroidaceae* and *Prevotellaceae*, *Desulfovibriaceae* increased, and the relative abundance of *Lachnospiraceae* decreased (Figure 6G).

In female mice, the four most important microbial phyla in the intestine were *Firmicutes*, *Bacteroidetes*, *Desulfobacteria*, and *Patescibacteria*. The relative abundance of *Firmicutes* increased and the relative abundances of *Bacteroidetes*, *Desulfobacteria*, and *Patescibacteria* decreased (Figure 6B). Compared with the control group, the relative abundance of *Clostridium* and *Bacilli* in the four most important microbial classes at the class level increased, and the relative abundance of *Desulfobacter* and *Bacteroidetes* decreased (Figure 6D); at the order level, the relative abundance of *Oscillospirales* increased, and the relative abundance of *Bacteroides*, *Desulfobacteria*, and *Lachnospirales* decreased (Figure 6F); Compared with the CF group, at the family level, the relative abundance of *Muribaculaceae* and *Prevotellaceae* increased, and the relative abundance of *Desulfovibriaceae* and *Lachnospiraceae* decreased (Figure 6H).

### 3.5. Diversity Analysis of Intestinal Microbial Flora in Male and Female Mice

In order to study the effect of PVC on the abundance and diversity of the intestinal microbiota in mice, we analyzed the alpha diversity of the intestinal microbiota. Compared with the control group, the indexes of the PVCF group and PVCM group decreased and had no significant effect (*p* > 0.1), indicating that continuous administration of microplastic PVC for 30 days caused a downward trend in the richness and uniformity of intestinal microorganisms in normal mice, but the difference was not significant (Table 3).

**Figure 7:**
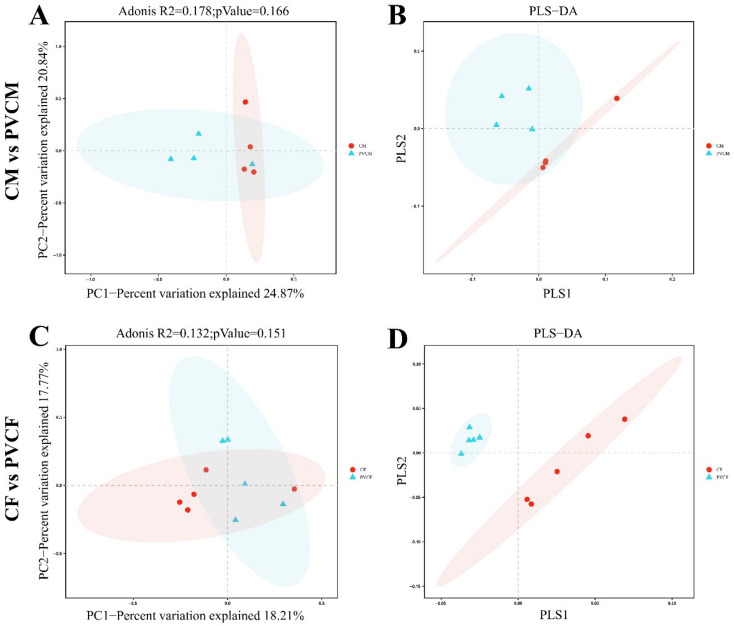
Beta diversity analysis of the gut microbial community of mice exposed to microplastics. (**A**) Adonis graph of CM and PVCM; (**B**) PCoA graph of CM and PVCM; (**C**) Adonis graph of CF and PVCF; (**D**) PCoA graph of CF and PVCF.

In order to compare the similarities between different samples in terms of species diversity, beta diversity analysis was performed on the gut microbiota. In the PCoA analysis, there was no significant difference between the PVCM group and the CM group (R^2^ = 0.178, *p* = 0.166) (Figure 7A). PLS-DA test showed that there was an intersection area between the CM group and the PVCM group, indicating that the composition of intestinal microflora in the CM group and PVCM group was highly similar (Figure 7B). PCoA analysis showed that there was no significant difference between the intestinal flora of mice in the PVCF group and CF group (R^2^ = 0.132, *p* = 0.151) (Figure 7C). In the PLS-DA test, CF was completely separated from the PVCM group along the PLS1 axis, and the similarity in gut microbiota composition between the CF group and the PVCF group was low (Figure 7D). The results showed that the pollution of PVC microplastics had a greater impact on the similarity of intestinal microbial flora in female mice.

### 3.6. Functional Prediction Analysis of Intestinal Microflora in Male and Female Mice

**Figure 8:**
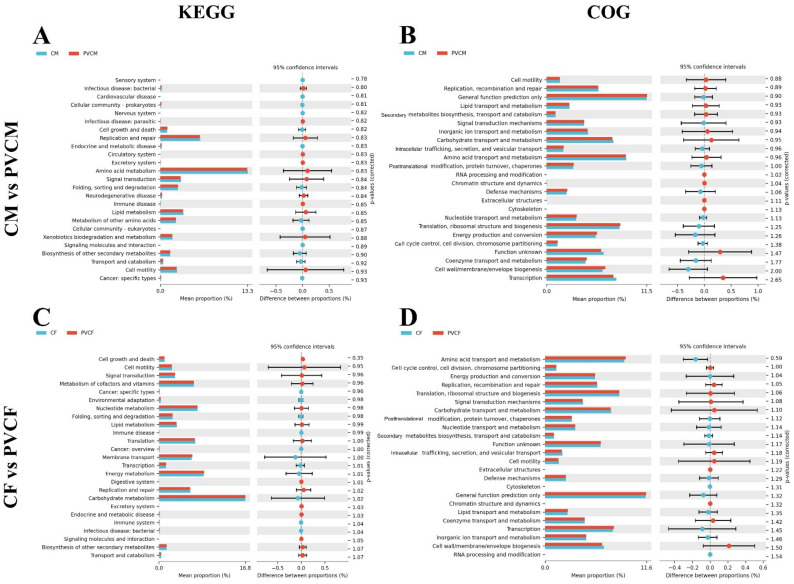
KEGG functional difference analysis and COG functional composition analysis in male and female mice. (**A**) KEGG Level 2 histogram of CM and PVCM; (**B**) COG histogram of CM and PVCM; (**C**) KEGG Level 2 histogram of CF and PVCF; (**D**) COG histogram of CF and PVCF.

In order to observe the differences and changes in functional genes in metabolic pathways and the composition of microbial communities between different groups of samples, we conducted KEGG functional difference analysis and COG functional composition analysis. In the KEGG functional difference analysis, the relative abundance of amino acid metabolism, replication and repair, and signal transduction metabolic pathways in the PVCM group decreased, and the pathways with more relative abundance increased were the metabolic pathways of biosynthesis, transport, and catabolism of other secondary metabolites. In the analysis of COG functional composition, compared with the CM group, the relative abundance of amino acid transport and metabolism, functional genes of transcribed functional genes decreased in the PVCM group, and the relative abundance of cell wall/membrane/membrane structure biosynthesis and signal transduction mechanisms increased (Figure 8A,B).

In the KEGG functional difference analysis, the relative abundance of replication and repair, cell activity, and vitamin metabolic pathways in the PVCF group was significantly reduced, while the more obvious pathways were mainly the biosynthesis, transport, and catabolism of other secondary metabolites. In the analysis of COG functional composition, compared with the CF group, the biosynthesis of cell wall/cell membrane/membrane structure, the relative abundance of functional genes of signal transduction mechanism decreased, and the relative abundance of functional genes involved in amino acid transport and metabolism, and transcription increased in the PVCF group (Figure 8C,D).

## 4. Discussion

### 4.1. Effects of Microplastic PVC on the Reproductive System of Male Mice

In recent years, there has been a notable increase in focus on the influence of microplastics on the ecological cycle. This has led to growing awareness of the potential risks associated with the constant accumulation of microplastics in organisms. The testis is the most crucial organ in the male reproductive system and is responsible for sperm production. The normal production of sperm in male mice is an essential component of maintaining the normal physiological function of the reproductive system and the reproduction of mice. In 2023, studies revealed the presence of microplastics in the testicles and semen of human males, with polyvinyl chloride (PVC) identified as the predominant polymer. The concentration of microplastics in the testes was found to be higher than that in the semen. However, the experiment did not reveal any discernible adverse effects of microplastics on the human body [17].

The potential toxic mechanisms of microplastics on the mammalian reproductive system caused by microplastic exposure have yet to be fully evaluated. The present study offers certain data and theoretical support for the evaluation of the effects of microplastics PVC on the reproductive system of male and female mice. This experiment also has certain limitations; that is, when mice were fed in groups, it did not take into account the influence of social hierarchy among mice in each group on the experimental results. It is hoped that this problem can be taken into account in future studies and that the intake of microplastics can be precise for each mouse to reduce the impact of unnecessary errors on the experiment.

In this experiment, the microstructure of male mouse testis slices exposed to PVC was observed, and the effect of PVC on the structure of the male reproductive system was compared and analyzed. Furthermore, the morphology of the seminiferous tubules in the testicular tissue of male mice exposed to microplastic PVC also underwent alterations, with a notable proportion of tubules exhibiting an oblong shape. The results demonstrated a notable increase in the cross-sectional diameter and cross-sectional area of the seminiferous tubules. It has been demonstrated that male mice exposed to 500 mg of PVC exhibit a notable reduction in the diameter of seminiferous tubules [18]. The administration of mice to polystyrene microplastics (PS) at a dose of 100 μg/L to mice resulted in a notable reduction in the lumen diameter of the seminiferous tubules [19]. These findings suggest that prolonged exposure to microplastic particles may result in notable alterations in the lumen diameter of the seminiferous tubules in male mice. Additionally, the junctions between seminiferous tubules exhibited compromised integrity, with a minority displaying looseness. Disordered spermatogenic cells were readily identified within the seminiferous tubules, exhibiting notable morphological alterations, and a limited number of connections between spermatogenic cells were observed to have been compromised. These findings are consistent with those of Jin et al., who observed that exposure to low-dose (10 μg/L) microplastic particles resulted in the arrangement of spermatogenic cells in the mouse testis becoming disordered, with the presence of multinucleated spermatogenic cells in the lumen of seminiferous tubules and even the detachment of spermatogenic cells from the basement membrane. However, when mice were exposed to a high dose of microplastic particles (100 μg/L), the results demonstrated that the arrangement of spermatogenic cells at all levels in the seminiferous tubules was disordered, and the spermatogenic cells detached from the basement membrane [20]. The study also indicated that with an increase in the dose of microplastic particles administered to mice, the arrangement of spermatogenic cells at all levels in the spermatogenic tubule increased, and the degree of cavity in the testicular tissue also increased. Long-term exposure to microplastic particles of varying concentrations and diameters has been demonstrated to exert deleterious effects on the quality of mouse sperm. These effects include a significant reduction in sperm motility and survival rate as well as alterations in sperm morphology and structure. Additionally, the incidence of sperm malformation has been shown to increase considerably in the presence of these particles [21,22,23]. Ingestion of 5 µm microplastics after 36 days of environmental exposure has been demonstrated to significantly reduce the number of live sperm in the epididymis and increase the rate of sperm malformation. Furthermore, the abnormal sperm quality observed in mice has been shown to be closely related to the Nrf2/HO-1/NF-κB pathway [24]. The results of these studies indicate that spermatogenic cells and spermatozoa excreted by male mice exposed to microplastic particles are affected by the physiological toxicity of microplastics. The aforementioned results indicated that microplastic PVC could induce reproductive toxicity in the testicular reproductive system of mice.

### 4.2. Effects of Microplastic PVC on the Reproductive System of Female Mice

It has been demonstrated that ingestion of a specific quantity of microplastics ingestion can induce reproductive toxicity in female mice. The objective of this experiment was to investigate the impact of PVC microplastics on the microstructure of the female reproductive system. These findings indicated that ovarian development was hindered, the diameter and number of follicles at all levels were markedly diminished, the number of atretic follicles was increased, and the ovarian reserve of female mice was diminished. The results demonstrated that PVC and polystyrene (PS) microplastics elicited comparable outcomes. Polystyrene microplastics (PS) were observed to be absorbed by ovarian granulosa cells, leading to the pyrodeath of these cells through the NLRP3/Caspase-1 signaling pathway. This, in turn, induces ovarian inflammation and oxidative stress, reduces the number of follicles, and affects the normal development and quality of oocytes [6,25]. The oxidative stress induced by microplastics PVC affects the reproductive system and reproductive ability of female mice, and it has been proven that microplastics are related to endometriosis, infertility, and oxidative damage of follicles, which cause reproductive obstacles, such as pregnancy rate and productivity reduction in female mice [26,27]. Furthermore, the ingestion of high doses of PVC microplastics by female ducks for a period of two months has been demonstrated to cause reproductive toxicity, including damage to follicular development and premature ovarian failure, eventually resulting in reproductive disorders [28]. In a separate study, Wistar mice were exposed to 5 μm microplastics continuously for four estrous cycles. The results demonstrated a significantly higher accumulation of microplastics in the corpus luteum, and atretic follicles of mice were significantly higher than those of secondary follicles and antral follicles. Additionally, there was a notable reduction in the ovarian weight coefficient, alterations in follicle formation, an increase in the duration of the estrous cycle, and ultimately, impairment of ovarian function in mice [29]. The above studies show that microplastics can affect the growth, development, and maturation of ovarian follicles in mice at different stages of ovarian and follicle development; therefore, microplastics can have different effects on the ovarian follicles of mice.

The function of fallopian tubes is to facilitate the transport of gametes and embryos, thereby enabling natural conception and preimplantation development. The volume of the uterus and fallopian tube in mice was visibly reduced PVC, with the muscle tissue appearing thinner, the endometrial tissue demonstrating a lower level of porosity, and the epithelial cells exhibiting a disordered arrangement. It is noteworthy that alterations in fallopian tube epithelial cells resulting from PVC may potentially contribute to the disruption of normal embryonic development. It has been found that in many species, including mice, extracellular vesicles (oEVs) produced by their fallopian tube epithelial cells have been observed to bind to oocytes, sperm, and embryos in numerous species, including mice. This binding improves the fertilization process, prevents polyspermia, and contributes to embryo development [30]. Prostaglandins PGE and PGF are also produced by tubal epithelial cells. These prostaglandins regulate muscle contraction through prostaglandin receptors, thereby optimizing the microenvironment in the tubal lumen [31]. Therefore, the tubal folds of the PVCF group mice are reduced, the normal morphology and structure of the epithelial cells are altered, and the muscle layer is lax, which results in aberrant hormone secretion and confusion regarding smooth muscle contraction. This will not only affect the interaction between the gamete and fallopian tube but also lead to abnormal embryo development before implantation.

Microplastics ranging in size from 2 to 200 μm, such as polypropylene (PP), polystyrene (PS), and polyethylene (PE), have been found to invade through dietary entry into the bloodstream [32]. However, the specific mechanism by which microplastic PVC invades the uterus remains unclear. However, our findings indicate that the foodborne microplastic PVC may be a contributing factor. The results of this experiment demonstrated that the degree of uterine development in the PVCF group was significantly lower than that observed in the CF group. The uterine body in the PVCF group was found to be significantly reduced in size, with notable alterations to the epithelial cell structure and a considerable reduction in muscle thickness. These observations suggest that the growth and development of the uterus may have been inhibited, with potential implications for the contractile and peristaltic abilities of the uterus and the cell number per unit volume of the endometrium. Additionally, the endometrium was observed to be denser. Additionally, the reduced elasticity of the endometrium may impact the implantation of embryos, potentially reducing the likelihood of successful pregnancy in mice. Additionally, microplastics have been demonstrated to activate diverse signaling pathways, thereby inducing oxidative stress and fibrosis in the uterus. It has been demonstrated that polystyrene microplastics exert their effects by acting on the TLR4/NOX2 signaling axis, thereby inducing oxidative stress and activating the Notch and TGF-**β** signaling pathways. This ultimately leads to the development of uterine fibrosis, which is a hallmark of the characteristics of endometrial adhesion (IUA) and other related conditions [33]. The above discussion indicates that the uterus, fallopian tubes, and ovarian tissues of female mice exposed to PVC microplastic particles are affected by the reproductive toxicity of microplastics, which has an impact on the growth and reproduction of female mice.

The macroscopic effects of the reproductive toxicity of microplastics have prompted researchers to investigate their underlying mechanisms of action. A comprehensive understanding of these mechanisms could potentially inform strategies to mitigate the reproductive toxicity of microplastics in animals. To elucidate the mechanism of microplastics’ effect on male mice, it is essential to consider the role of reactive oxygen species (ROS). These species play a pivotal role in maintaining homeostasis of the male reproductive system, influencing processes such as spermatogenesis, sperm function, and fertilization. It has been demonstrated that microplastics can damage the integrity of the male reproductive system blood−testicular barrier (BTB), resulting in testicular malformation and spermatogenic dysfunction. This is achieved through the ROS-associated Nrf2/HO-1/NF-κB, p38 MAPK, and MAPK/Nrf2 signaling pathways [34].

### 4.3. Correlation Analysis of Intestinal Microbiota and Reproductive System Tissue and Organ Indexes in Male and Female Mice

The co-evolution of intestinal microbes and mammalian hosts has resulted in the formation of a unique symbiotic relationship whereby intestinal microbes play a pivotal role in the host’s energy metabolism, metabolic signals, immune system development, maintenance of intestinal barrier integrity, etc. The impact of changes in microbial flora on the reproductive endocrine system may influence reproductive outcomes [35,36]. The amount of genetic information in the intestinal microbiome is over 100 times higher than that in the human genome and is intimately associated with a range of biological processes and disease states [37]. The gut microbiome has the capacity to regulate neurophysiological processes by modifying immune, endocrine, reproductive, and neural signaling pathways via the “gut-brain axis” [38]. The gut microbiome and its metabolites can act as signaling molecules, facilitating communication between the gut, liver, brain, and reproductive tract [39]. It has been demonstrated that the gut microbiome may exert an indirect influence on the reproductive axis through its impact on metabolism and may exert a direct effect on the reproductive axis by promoting liver-enterohepatic circulation and increasing estrogen responsiveness, thereby regulating reproduction through the modulation of steroid hormones [40]. Ding et al. demonstrated that the gut microbiome affects sperm generation. Furthermore, a high-fat diet group can induce phenotypically variable gut microbiome dysbiosis in mice through fecal microbiota transplantation (FMT), which can lead to male infertility, where male infertility caused by methionine has become a major global reproductive health problem [41].

This section primarily addresses the functional and regulatory mechanisms of intestinal flora associated with reproductive system damage in male and female mice induced by PVC. To date, there has been a paucity of studies and analyses examining the potential correlation between microplastic PVC and the intestinal microflora and reproductive system of mice. Given the possibility of differing effects of PVC on male and female mice, Spearman correlation analysis was employed to further examine the relationship between intestinal microflora imbalance and reproductive dysfunction in male and female mice induced by microplastics, thereby offering new avenues for future research.

In male animals, alterations in the composition of gut microbiota are strongly linked to the development of reproductive disorders. A substantial body of evidence from numerous studies has substantiated the assertion that intestinal microbial disorders can exert an influence on the IL-17A signaling pathway of the testis. Furthermore, there is compelling evidence to suggest that intestinal microbial flora plays a pivotal role in the etiology of male reproductive disorders [42]. The results of the Spearman correlation analysis of male mice indicated that PVC exposure could result in an imbalance in the intestinal flora and subsequent testicular injury, showing that the intestinal *Actinobacteria*, *Corynebacteriales*, and *Staphylococcaceae* in male mice exhibited a significant positive correlation with damage to the male reproductive system. *Actinobacteria* are Gram-positive, multi-branched, non-motile, non-spore-producing anaerobic bacteria. It primarily comprises anaerobic bacteria (*Bifidobacteria*, *Propionibacteria*, and *Corynebacteria*) and aerobic bacteria (*Streptomyces*) [43]. Despite representing a relatively minor proportion of intestinal symbiotic bacteria, Actinomycetes are of pivotal importance in maintaining intestinal homeostasis. They are postulated to be instrumental in regulating intestinal permeability, the immune system, metabolism, and the gut-brain axis [44]. The notable increase in Actinobacteria mitigates the harm caused to the male reproductive system by microplastics by prompting regulatory T cells to regulate immune inflammation and autoimmune responses [45,46]. *Staphylococcus* is a bacterial species that plays a symbiotic role in the body of animals and can also behave as an opportunistic pathogen. It has been demonstrated that *staphylococcal* infection may result in the mediation of local or systemic inflammation in reproductive tissues, which is highly correlated with reproductive injury and infertility. When the products of *Staphylococcus* are recognized by toll-like receptors (TLRs) and G-protein-coupled receptors in testicular cells and epididymis tissues as ligands, they can activate inflammatory signaling pathways and induce local or systemic inflammatory responses through the release of pro-inflammatory mediators, while the adaptive immune response of the body may aggravate the severe injury induced by inflammation. This results in a decline in sperm quality and dysfunction of testicular neuroendocrine mediation in male animals, which ultimately damages fertility [47]. Although *Staphylococcus* is one of the most virulent bacteria and a significant contributor to infertility in both men and women, the mechanisms underlying its action remain poorly understood.

In female animals, normal intestinal microbes can affect the reproductive system by interacting with estrogen, androgen, insulin, and other hormones. Conversely, an imbalance in the intestinal flora can lead to a variety of diseases of the female reproductive system, including pregnancy complications, polycystic ovary syndrome (PCOS), and endometriosis. [35]. The results of this experiment indicate a positive correlation between the presence of *Fusobacteriota* and *Veillonellales-Selenomonadales* in the intestinal tract of female mice were positively correlated with the damage of the reproductive system (*p* < 0.01). *Fusobacteriota* has been isolated from tumor samples and is associated with local lymph node metastasis. The microorganism in *Fusobacteriota* can produce butyric acid from carbohydrates and mucin, and an increase in its abundance contributes to an increase in the concentration of short-chain fatty acids in the intestinal chyme. Short-chain fatty acids are the most significant metabolites produced by intestinal flora. It plays a pivotal role in maintaining the intestinal epithelial cell barrier and alleviating inflammatory bowel disease [48,49].

In conclusion, it can be proposed that the imbalance of intestinal microflora in male and female mice exposed to foodborne microplastics (PVC) may be associated with reproductive damage. This section is dedicated to the analysis of reproductive toxicity resulting from the imbalance of intestinal flora in mice of different sexes caused by PVC microplastics. This study provides a comprehensive summary and analysis of the intestinal flora that are strongly associated with the reproductive system at a single level, offering novel insights for future research on the impact mechanism of microplastics on biological intestinal microflora and the reproductive system.

It has been demonstrated that the activation signaling pathways of microplastics differ between males and females. It has been demonstrated that exposure to microplastics can result in an elevation of ROS levels, which, when they exceed a certain threshold, can lead to the induction of damage to the reproductive system [6,50]. It has been demonstrated that exposure to microplastics can affect reproduction by increasing the activation of signaling pathways related to ROS. OS can result in alterations in the expression levels of a range of proteins and inflammatory factors, thereby activating associated signaling pathways, ultimately leading to testicular lesions, ovarian dysfunction, and other adverse effects on the reproductive system. In females, microplastics contribute to female reproductive dysfunction through ROS-related Wnt/β-Catenin and NLRP3/Caspase-1 signaling pathways, such as granulosa cell apoptosis, decreased ovarian volume, and decreased oocyte production [34].

### 4.4. Effect and Analysis of Microplastic Polyvinyl Chloride on Small Intestine Microstructure of Male and Female Mice

The intestinal tract is the largest digestive and absorptive organ in the animal body. The integrity of the intestinal microstructure is a fundamental determinant of the efficacy of nutrient absorption in mice. A number of distinctive structures within the small intestine are intimately associated with nutrient absorption, including villi, microvilli, and crypts. The width, height, and crypt depth of the small intestine villi are all important indicators for measuring the absorption and digestive function of the small intestine. Among these, the ratio of villi height of the small intestine to crypt depth can effectively reflect the absorption function of the small intestine, with a higher value indicating a faster digestive and absorption rate [51]. The stem cell population located at the base of the intestinal crypt serves as the source of all intestinal epithelial cells. Their progeny migrate upward from the crypt to the villi, ultimately reaching the lumen at the tip of the villi tip [52]. A disruption to the division of stem cells at the base of the crypt will impact the intestinal repair ability, subsequently affecting the production and secretion of antimicrobial substances by intestinal cells. This will result in a weakened antibacterial ability of the intestinal mucosa and a decline in the digestive and absorptive capabilities.

The effects of PVC microplastics on the microstructure of the small intestine in both male and female mice were as follows: there was a decrease in intestinal villus height, destruction of villus structural integrity, a decrease in villus surface area, an increase in crypt depth, and a significant decrease in the ratio of villus height to crypt depth. These findings align with those of previous microplastic PVC studies, including the observation of shortened and swollen intestinal villi of perch, structural fusion and apical collapse, leukocyte infiltration, congestion, and loss of villi and crypt cells in juvenile intertidal hairtail fish [10,53]. These alterations provide a rationale for the hypothesis that microplastics will result in the destruction of the intestinal structure of mice, affect intestinal cell division, impair the anti-damage and digestive and absorptive capabilities of the small intestine, and cause dysfunction of the small intestine of mice.

### 4.5. Effects of Microplastic PVC on Intestinal Microflora in Male and Female Mice

The stability of the structure and function of the intestinal microflora is beneficial to the body in several ways. Firstly, it enables the body to obtain energy from digested food. Secondly, it prevents colonization by pathogens. Thirdly, it regulates immune function. Finally, it strengthens the biochemical barrier of the intestine. A disruption in the intestinal flora can result in the onset of immune-mediated inflammation and the impairment of the intestinal mucosal barrier. One of the mechanisms underlying intestinal inflammation is the disturbance of the immune system. In this study, high-throughput sequencing was conducted on the intestinal contents of mice in the ileum, cecum, and colon to elucidate the impact of intestinal microorganisms on PVC microplastic pollution. The aim of this study was to investigate whether the effects of PVC microplastics on the intestinal microflora of mice differ depending on the sex of the animal.

The beta diversity analysis and the PLS-DA analysis of the PVCF group revealed a complete separation from the control group, with a greater divergence than that observed in the PVCM group, which exhibited less separation. In the characteristic taxonomic analysis, compared with the control group, the microbial species of female mice exhibited an increase at all levels except for the family and genus levels, while the microbial species of male mice demonstrated a decrease, except for the aforementioned increase at the order and family levels. These findings indicate that the impact of PVC microplastics on the intestinal microflora of mice exhibits sex-specific differences.

In the analysis of species composition at each level, the majority of the microflora with a high relative abundance in male and female mice exhibited an inverse trend. The predominant phyla in the normal intestinal flora of mice were *Bacteroidetes* and *Firmicutes*, with *Actinobacteria*, *Proteobacteria*, and other phyla. *Firmicutes*, one of them, is one of the principal bacterial phyla in a healthy gut. It has been demonstrated that intestinal *Firmicutes* and their metabolites possess health-promoting functions when induced by dietary fiber [54]. An increase in its abundance may be associated with an enhancement in the anti-inflammatory effect of the intestinal tract and an improvement in the integrity of the intestinal barrier. Conversely, a reduction in its relative abundance may result in a diminished anti-inflammatory effect in mice that will be weakened [55]. At the phylum level, the relative abundance of *Firmicutes* in the intestinal microbes of mice in the PVCM group was reduced, which was consistent with the results of the other two experiments [56,57]. In contrast to the PVCM group, the relative abundance of Firmicutes increased in the PVCF group, indicating that the intestinal anti-inflammatory capacity of female mice was enhanced by PVC. Short-chain fatty acids represent the most significant metabolites produced by the intestinal flora. They play a pivotal role in maintaining the integrity of the intestinal epithelial cell barrier and alleviating inflammatory bowel disease (IBD) [58]. The relative abundance of Firmicutes has been demonstrated to enhance the host’s absorption of short-chain fatty acids. Consequently, the observed reduction in Firmicutes’ relative abundance within the PVCM group may be indicative of a diminished capacity for short-chain fatty acid absorption, which could ultimately result in a reduction in overall energy intake. This, in turn, may contribute to the pathogenesis of inflammatory bowel disease. The *Bacteroidetes microbiome is* the largest intestinal microbiome and plays a pivotal role in host food digestion and nutrient absorption. It is involved in a multitude of functions, including carbohydrate, bile acid metabolism, and steroid metabolism [59]. It has been demonstrated that an increase in the relative abundance of *Bacteroides* is associated with an increase in the oxidative stress level in the host body. Conversely, a reduction in its relative taxonomic abundance has been linked to an increased risk of developing metabolic syndrome, coronary heart disease, non-alcoholic steatohepatitis, and other diseases [60,61]. The proportion of Bacteroides and non-Bacteroides in the intestinal microflora of mice exposed to PVC will increase, which will affect the body’s carbohydrate metabolism and fat metabolism rates [62]. Concurrently, research has demonstrated that an increase in the relative abundance of *Bacteroidetes* will elevate metabolite levels, thereby triggering an inflammatory response and compromising the integrity of the intestinal mucosal barrier. Furthermore, certain bacterial groups within the Bacteroidetes phylum are involved in the release of toxic substances during protein decomposition. These metabolites can act on inflammatory response pathways, affect the body’s barrier function, and exacerbate the inflammatory response of the body [63,64]. The results of this experiment demonstrated that the relative abundance of *Bacteroides* in the PVCM group increased at the phylum, class, and order levels. This was in contrast to the results observed in the PVCF group, indicating that exposure to PVC could significantly enhance oxidative stress levels and exacerbate the inflammatory response in male mice. Additionally, it was observed that this exposure could affect the digestive and absorptive abilities of the intestine and potentially elevate the risk of enteritis.

A change in the ratio of *Firmicutes* to *Bacteroidetes* (F/B) is regarded as an indicator of an imbalance in intestinal flora. An increase or decrease in F/B ratio is perceived as an ecological imbalance. The former is typically observed in individuals with obesity, whereas the latter is frequently observed in individuals with inflammatory bowel disease (IBD). However, alteration in the ratio is also influenced by other factors, and the outcome is not definitive [65]. The sequencing results indicated that, in contrast to the PVCF group, the F/B ratio decreased in the PVCM group, which is consistent with the findings of Sun et al. However, other researchers have reported different results, with no change in the F/B ratio or even an increase in some cases [66,67].

*Desulfurobacteria* are anaerobic bacteria that can harm the intestinal tract. *Desulfurvibrio* is distinguished by its capacity to utilize lactic acid, pyruvate, ethanol, and certain fatty acids as carbon and energy sources, facilitating the reduction of sulfate to hydrogen sulfide. Endogenous hydrogen sulfide has been demonstrated to exert toxic effects on intestinal epithelial cells, potentially leading to a range of adverse outcomes, including intestinal sensitivity, intestinal leakage, and abdominal pain. Clinical studies have corroborated the assertion that an increase in the number of *Desulfurvibrio* is a significant indicator of colorectal cancer and that it can facilitate the proliferation of cancer cells [68]. The increase in desulfurizing bacteria in the PVCM group was contrary to the results observed in the PVCF group, which also demonstrated that intestinal damage in male mice was more severe than that in female mice.

The role of microorganisms in the gut is to exert a number of effects on immunity, the absorption of nutrients, and even the metabolism of enzymes [69]. In functional prediction analysis and KEGG analysis, PVC significantly reduced the relative abundance ratio of signal transduction pathways, which was closely related to environmental information processing. This indicates that PVC microplastics would impair the body’s function in environmental information processing and that the metabolic efficiency of the PVC group decreases [70]. The relative abundance of functional genes in male and female mice did not overlap; however, the relative abundance ratio exhibited a notable decline across the board. A clear distinction was observed between the male and female mice in the COG classification system. In the PVCF group, there was a notable decrease in the relative abundance of functional genes involved in the biosynthesis of the cell wall/membrane/membrane structure, as well as in signal transduction mechanisms. Conversely, there was an increase in the relative abundance of functional genes associated with amino acid transport and metabolism as well as transcriptional processes. The upregulation or downregulation trend of PVCM for these functional genes were opposite to that of PVCF. Amino acid transport and metabolism can facilitate the host’s metabolism of a substantial number of nutrients that are not metabolized by the host. The amino acid transport and metabolism of PVCF are enhanced, while PVCM is diminished, suggesting that the digestion and absorption of fat and amino acids in the PVCF group are augmented, whereas those in the PVCM group are diminished. The generative ability of microorganisms and molecules in the PVCM group was observed to increase, while that in the PVCF group was found to decrease [71].

## 5. Conclusions

In conclusion, the results of this study demonstrate that ingestion of PVC microplastics results in structural damage to the reproductive system, impairment of the intestinal barrier, and disruption of intestinal microflora in both male and female mice. It is noteworthy that sex differences were observed in the effects of PVC microplastics on the intestinal microflora of mice, with contrasting impacts on both the intestinal microflora and function. This study provides a foundation for further investigation into the toxicity of microplastics on the intestinal and reproductive systems of mice of different sexes exposed to microplastics; however, the potential effects and harms of microplastic PVC on mammals of different sexes remain largely unstudied. It is notable that the comprehensive toxicity investigations of microplastics in the actual ecosystems are currently limited. It is hoped that this experiment will provide a foundation for future research on the systemic effects of various types of microplastics on animals of different sexes, attract greater attention from researchers to the potential threats caused by microplastic exposure, and facilitate the acquisition of more comparative and supplementary experiments.

## Figures and Tables

**Table 1:** Effects of microplastic PVC on intestinal structure in male and female mice (n = 6).

Items	Object	CF	CM	PVCF	PVCM
Duodenum	Villus wide/µm	57.66 ± 2.23	58.22 ± 4.15	66.06 ± 5.75	50.82 ± 4.00
	Villus height/µm	814.97 ± 29.67 ^a^	696.08 ± 20.38 ^b^	648.08 ± 28.06 ^c^	575.75 ± 27.58 ^d^
	Crypt depth/µm	117.42 ± 4.21 ^ab^	101.18 ± 3.88 ^b^	159.91 ± 4.97 ^a^	128.62 ± 4.37 ^a^
	Villus height/Crypt depth	6.95 ± 0.48	6.89 ± 0.27	4.05 ± 0.12	4.48 ± 0.15
Jejunum	Villus wide/µm	69.14 ± 8.77 ^a^	60.93 ± 4.15 ^a^	58.39 ± 9.72 ^b^	51.46 ± 4.00 ^b^
	Villus height/µm	745.91 ± 34.68 ^b^	796.88 ± 12.42 ^a^	614.32 ± 28.55 ^d^	708.87 ± 18.48 ^c^
	Crypt depth/µm	110.12 ± 4.77 ^b^	104.44 ± 3.66 ^b^	140.81 ± 8.54 ^ab^	163.88 ± 6.49 ^a^
	Villus height/Crypt depth	6.79 ± 0.53	7.64 ± 0.35	4.38 ± 0.35	4.33 ± 0.14
Ileum	Villus wide/µm	62.55 ± 4.71 ^a^	55.12 ± 4.15 ^ab^	46.71 ± 4.54 ^b^	41.17 ± 4.00 ^b^
	Villus height/µm	589.58 ± 23.23 ^b^	715.89 ± 34.63 ^a^	482.72 ± 19.27 ^c^	575.66 ± 28.44 ^b^
	Crypt depth/µm	112.54 ± 5.23 ^b^	102.64 ± 4.00 ^b^	132.46 ± 4.40 ^ab^	149.61 ± 5.71 ^a^
	Villus height/Crypt depth	5.25 ± 0.40 ^a^	6.99 ± 0.52 ^a^	3.65 ± 0.23 ^b^	3.85 ± 0.24 ^b^

Different letters on the inter-digit scale indicate significant differences (*p* < 0.05), lowercase letters indicate significance level a = 0.01, and the same letter or no letter indicates no significant difference (*p* > 0.05).

**Table 2:** Effects of microplastic PVC on the number of microbial species at all levels in male and female mice (n = 5).

Sample	Phylum	Class	Order	Family	Genus	Species
CF	12.2 ± 0.45 ^A^	17.6 ± 0.55 ^a^	36.8 ± 2.17	55 ± 3.94	106.2 ± 6.10	32.6 ± 4.93
PVCF	11.8 ± 0.45 ^ab^	17.2 ± 0.84 ^a^	37.2 ± 2.59	56 ± 4.30	106.4 ± 9.40	31.2 ± 3.83
CM	10.75 ± 0.96 ^B^	16.25 ± 0.50 ^b^	35 ± 0.82	55 ± 3.56	107 ± 1.63	30.25 ± 2.06
PVCM	11.5 ± 0.58 ^b^	17 ± 1.41 ^ab^	36 ± 1.63	53.75 ± 6.08	105.75 ± 8.46	34.5 ± 4.43

Different letters in the same column indicate significant differences (*p* < 0.05), and uppercase letters indicate significance level A = 0.05; lowercase letters indicate significance level a = 0.01, and the same letter or no letter indicates no significant difference (*p* > 0.05).

**Table 3:** Effects of PVC on Alpha diversity of the gut microbiota in male and female mice (n = 5).

Sample	Feature	ACE	Chao1	Shannon	Simpson
CF	802.60 ± 89.16 a	810.08 ± 91.55 a	810.98 ± 92.02 a	7.80 ± 0.16	0.99 ± 0.01
CM	750.75 ± 104.10 ab	759.55 ± 103.86 ab	759.21 ± 103.46 ab	7.54 ± 0.68	0.98 ± 0.02
PVCF	753.40 ± 106.89 ab	757.50 ± 109.30 ab	757.75 ± 110.24 ab	7.76 ± 0.33	0.99 ± 0.00
PVCM	686.25 ± 84.68 b	688.47 ± 85.79 b	687.64 ± 85.42 b	7.49 ± 0.44	0.980.01

Different letters of the inter-label in the same column indicate a significant difference (*p* < 0.05), lowercase letters indicate significance level a = 0.01, and the same letters or no letters indicate no significant difference (*p* > 0.05).

## Data Availability

The original contributions presented in the study are included in the article, and further inquiries can be directed to the corresponding authors.
